# A Statement of the Early Symptoms Which Lead to the Disease, Termed Water in the Brain; with Observations, &c.

**Published:** 1816-04

**Authors:** 


*A Statement of the early Symptoms which lead to the disease, termed* WATER IN THE BRAIN; *with Observations, &c.* By G. D. YEATS, M. D. 8vo. pp. 114. London, 1815.

AN attention to the early symptoms and insidious approaches of Hydrencephalus, becomes a matter of great importance from their equivocal nature, and their being common to some other diseases, whereby the hasty examiner is deceived till the fatal effusion on the brain takes place. Although Dr. Yeats, as well as some others, considers hydrencephalus as occasionally owing to *primary* morbid action in the brain, and especially as resulting from mechanical violence there, yet in the vast majority of cases, he traces it to derangement of function or structure in other parts, and particularly the abdominal viscera. Rush, Quin, and Whytt seem to have not suspected this; and although Fothergill notices the morbid state of the bowels, the remark led to no curative indication. Cheyne was the first to direct our attention to the state of the abdominal viscera. Dr. Yeats asserts, that when hydrencephalus results from external violence, yet the internal causes "are seated in the digestive organs, the functions of which become deranged with various symptoms; and of this derangement, the *hepatic* powers, from their great importance, largely partake."

In the very first commencement of the symptoms, an occasional languor, as if arising from fatigue, with intervals of activity is observed—the healthy appearance of the countenance diminishes, evinced by transient paleness and collapse of the features; dark coloured line under the eye, and dullnes of that organ. The softness and pliability of the skin diminish with consequent harshness and increased heat on the surface. The appetite becomes capricious; there is occasional thirst; the bowels are torpid; the tongue white; the pulse is unaltered; the urine occasionally high coloured. When by any domestic purgative, the bowels are moved at this time, the stool is scanty, and unusually firm, without much discolouration; though on attentive examination, it will be perceived that diseased secretion has taken place in the bowels.

The fæces will be sometimes too light in colour, sometimes greenish and shining. Uneasiness, rather than pain, will now sometimes be complained of in the head, with external soreness. Examination at this time, will detect a puffiness or fulness over the centre of the stomach, extending to the navel, with uneasiness on pressure; all which symptoms, excepting the costiveness, are not permanent, but transitory. The sleep is disturbed by uneasy motions; in short, the child is merely said to be unwell from some improper food, &c. though this irregular excitement, and vacillating state, very frequently lead to the next chain of more manifest morbid actions terminating in hydrencephalus. While these symptoms are impending too, the children are generally gifted with a precision of ideas and quickness of apprehension much beyond their years, and thus become more interesting objects to their friends, while their loss is more severely felt. The duration of this prelusive state of commencing disease, will depend on many circumstances, and is, therefore, extremely uncertain, being sometimes rapid, sometimes protracted. As constipation is the prominent symptom, and as domestic laxatives are occasionally administered, the ultimate stage of the disease is sometimes warded off for a time, or even in good constitutions, and favourable circumstances, the morbid action will be entirely removed. In a great majority of cases, however, this is not sufficient, and it becomes necessary to check the forming disease by the combination of an *alterative* with the purgative, in such a way, that the diseased secretions may not only be removed, but corrected.

"The good to be derived," observes our author justly," "does not depend upon many evacuations produced by a strong purgative, but by a gradual restoration of healthy action, to the glands of the intestines, and to the alimentary canal."

Hence a *change* of purgative is often necessary, since some kinds act more on one set of glands, and others on a different set.

"I have commonly given,', says Dr. Y. "the compound extract of colocynth with calomel, or the latter with aloes, rhubarb, or scammony, twice or thrice in the twenty-four hours."

In other circumstances, these might be too irritating, but here the bowels have an inveterate torpidity requiring constant attention. It is not necessary, however, in this stage, that the mercury should take any constitutional effect. The puffiness and fulness about the region of the stomach, Dr. Yeats is disposed to attribute to a distension of the *duodenum;* and much theoretical speculation is here introduced to shew how a primary affection of this second stomach may produce engorgement of the liver, a disordered state of the bowels, and finally hydrencephalus from sympathy with these organs. This hypothesis, however, we shall not dilate upon. We are quite confident, that whether as primary or secondary affections, the disordered states of the abdominal viscera must be corrected in hydrocephalus or its precursory symptoms, and, therefore, we shall not dispute about the particular viscus in the latter class, which is the original seat of the disease. We would just hint, nevertheless, that the vascular action of the brain should be attended to, during our operations on the digestive organs; and that we should by all possible means divert the sanguineous determination to some less important part, than the cerebral mass. Dr. Yeats says, he has made it an established rule of practice, to join purgatives with the calomel; but we do not see how this idea accords with his former recommendation of employing calomel as an *alterative*. We ourselves are of opinion, that however necessary may be cathartics, the mercurial preparations are also necessary to promote healthy secretions from the liver and intestinal canal; and this object they will effect better by remaining some time in the primæ viæ, than by being hurried through that tract in combination with "other purgative ingredients." This seems to be acknowledged by our author himself, who, a page or two farther on, remaks, that

"It is indeed remarkable how easily the bowels very soon assist themselves in passing off their contents, *after the glands have been excited to healthy action*, thus aiding the more healthy bile now poured into the intestines from a better conditioned liver." 57.

When the disease arrives at the point of great excitement on the cerebral vessels with effusion, the greater number of cases sink hopeless into the grave; yet even here, Dr. Y. does not consider them as to be always entirely despaired of. The occasional languor wears now more the appearance of permanent lassitude; the returns of activity diminish; the child seeks a recumbent posture; the countenance has a permanently unhealthy appearance; the darkness under the eye has a deeper colour; the febrile excitement is more marked; with dry skin; flushed cheeks; transient pains in the head, more or less acute. The pulse now becomes occasionally quickened, not particularly irregular; periods of drowsiness supervene; the bowels become more obstinately costive. The stools are morbid in appearance and smell; sometimes a glutinous mass intermixed with dark lumps of fæces; at others, a mixture of deep green with matters similar to yeast. Sickness, nausea, and vomiting are now troublesome. The puffiness and fulness about the stomach subside, "one part of the morbid actions having yielded to others of a more violent nature." A giddiness, with an unpleasant cloudiness in the sight is complained of, and light is painful. The urine is various. The appetite fails; the thirst is troublesome; the tongue is white, and inclining to become dry. The complaint is still manageable, but every hour is precious, and a few moments lost are scarcely to be recovered. The plan must now vary according to circumstances. If there be much vascular action, general or topical blood-letting, or both, must be had recourse to, "nor need we be timid respecting this evacuation, from a false principle, that the disease arises from debility." 62. It may be properly urged here, that general should not supersede the necessity of local blood-letting, since the inordinate action of the *particular* vessels giving rise to the effusion is frequently independent of the general increased vascular action. *Vide Dr. Parry's Elements of Pathology, lately reviewed.*

"It is on this principle that topical abstractions of blood are so useful, aided by the powerful alterative effects of mercury. The fatal effusions in the brain will be hastened and increased by permitting this evident inflammatory tension to continue; and from the preternatural vascular activity which also exists in organs subservient to digestion, the application of leeches to the region of the stomach and liver, as well as to the head, will be found essentially useful." 65.

"For the same reason it is that the neutral salts, particularly the sulphate of potass, given twice or thrice a day, either in infus. rosæ, or saline draughts, are always preferred by me at this time to the resinous purgatives, never failing to give every night two or three grains of the submuriate of mercury." *ib.*

Dr. Y. asserts, that he has uniformly found in all inflammatory complaints, a combined exhibition of calomel and the neutral salts, in small divided doses, a most powerful antiphlogistic. Blisters, he considers as a matter of nice discrimination in their application, and that full evacuations should always be premised. The diet, of course should correspond with the above views of the disease.

"No spicy or spirituous stimulants, nor animal food, should enter into its composition."

When by pursuing this plan, the morbid symptoms give way, Dr. Y. thinks a *tonic* should be daily given, conjoined with an opening medicine that may insure regular evacuations. We humbly conceive, that the best tonic is the removal of the disease, and that Nature will crave for food as soon as she is capable of digesting and assimilating it; at least, this is the result of our observations in more diseases than—"Water in the Head."

The accession of the next and most dangerous state of the complaint is marked by a greatly increased degree of violence in the symptoms, and sufferings of the patient. The heat of the skin becomes more intense and harsh; the febrile accessions more violent and distressing; the head-ache more acute, with afflicting screams on the part of the child; the pupils of the eyes become preternaturally dilated, but contract irregularly on the application of light; there is occasional strabismus; double vision is complained of, with *error loci* of objects. Knitting of the eye-brows with countenance expressive of distress. For a few minutes there will be perfect silence and rest, with fixed steady stare of the eyes, and much dilatation of the pupil; when a sudden start will take place, with a loud scream and quick tossing of the arms over the head; frequent moaning; deep sighing. Sickness and vomiting; bowels most obstinately costive; the evacuations, when procured, scanty, ill-formed, and extremely offensive. When by active means, a mass is brought away, it is totally unlike fæces, being dark, yeasty, gelatinous, and smelling like sour grains with putrid matter. The tongue now becomes foul, sometimes brown and dry. Much thirst; no appetite; urine irregular; pulse very much so, both in the tone of the vibration, and flow of the blood; sometimes slow, sometimes quick, and intermitting with a tensive feel, until at last it sinks into a permanent sluggishness, ushering in its ultimate and fatal celerity. A dewy moisture settles upon the upper-lip and around the nose. A considerable wasting of the flesh has now taken place; the countenance is pallid and sunk, with a hollowness of the temples; blueness of the lips; attempts, but inability to cry, ending in a whining tone. The eye-lids half open and motionless; the eyes filmy and fixed with a peculiar stare. The circulation is extremely hurried; convulsions frequently take place; palsy supervenes; death, most commonly in one convulsive struggle, closes the scene!

The recovery of cases under apparently similar circumstances renders this condition, though deplorable, not absolutely hopeless, especially at the commencement of this —the last, stage. Every attempt should be made by repeatedly leeching the temples; by general bleeding, if the strength will permit; by blisters to the head, kept open to diminish the vascular action in the brain. To these must be added a diligent use both internally and externally, of mercury, which powerfully assists the other means in altering that morbid excitement constituting the disease. All this time, the bowels are to be daily emptied.

"And although," says Dr. Y. "there shall be every appearance that effusion has taken place, the patient not unfrequently recovers completely by the use of those medicines which carry off effused fluids, more especially when the constitution is under the influence of mercury." 78-9.

The circumstance of bleeding producing a state of the constitution more favourable to the mercurial action, is adverted to by our author; and the remark must be familiar to the readers of the New Medical and Physical Journal, where we have repeatedly stated it. With respect to diuretics, our author lays little stress upon them, unless the vascular action of the brain be previously reduced: they are, however, useful on the principle of counter irritation by increasing the secretion from other glands. The digitalis he considers a doubtful remedy. Dr. Y. here relates a successful case in his own practice, where effusion had actually taken place, "with widely dilated pupils; loss of sight; paralysis, &c." Mercury was very liberally used, both internally and externally; leeches were repeatedly applied to the head and to the epigastrium; the head was shaved; a blister applied over the whole of it, and kept open; to which were added, saline, diuretic, and purgative medicines. Although a very considerable quantity of mercury was used, no salivation took place; but as the recovery approached, the bowels were more easily acted on, and the evacuations exhibited a more healthy appearance.* The sight and ability to walk gradually returned. Dr. Y. thinks, that, in whatever portion of the organs subservient to digestion, whether the glandular or hollow viscera, the irritation commences, it will produce a *general* effect proportioned to its degree, and gradually excite into morbid action the contiguous parts, thus enlarging by a kind of irradiation, till the whole of the digestive organs are concerned, when will commence the cerebral irritation from sympathy.

"The liver, as one of the most important organs in every way, will partake most largely of this irritation, both by contiguity, if the irritating cause has not commenced in itself, and by sympathy; hence, as its secretion is the most obvious from its sensible qualities, we the more readily perceive the changes produced by this morbid state." 84.

When the irritation is once propagated to the brain, it is easy to see that by *reaction* a reflected exasperation of the original disorders will be the consequence. It is a matter of little import in what portion of the digestive organs the original derangement of action shall commence,

---

* A case of recovery from this disease is stated in Med. Memoirs, vol. xiv. of a child eighteen months old, where no salivation took place, although six ounces and a half of mercurial ointment were rubbed in, and thirty-six grains of calomel exhibited by the mouth.

in respect to the production of the hydrencephalic state; but the difference is a matter of importance with respect to the facility of the cure, inasmuch as the simple is more reducible than the combined irritation. It is also probable, that the impression made upon the brain by a portion only of the digestive organs being in a diseased state, will be more easily cured from a less intensity of cause, than when that disposition arises from the whole of the chylopoietic organs being under morbid action; "hence it is," Dr. Y. thinks, "that extremely severe cases have terminated favourably, when others, equally or less severe in appearance, have ended in death." 86.

In cases of long continued deranged functions of the digestive organs, previous to excitement of the cerebral vessels, portions of the abdominal viscera are almost always found diseased, as appears from dissection, and thus account for morbid evacuations.

"In whatever way that very important organ, the liver, may be affected, the affection will undoubtedly have, sooner or later, very considerable influence on the system at large, and not from diseased bile alone, though this is of much importance. When the peculiarity of its own circulation, and the great proportion of the mass of blood, both arterial and venous, occupied by it, are considered, a disturbance in any way of this quantity of fluid passing through it, must make a corresponding impression upon the whole circulation of the body, independent of those sympathetic irritations to which a living machine, from the intimate and vital connection of all its parts, is subject."

Dr. Yeats next points out the determination of blood to the head, which must be produced when any difficulty of transmission through the liver takes place, a circumstance previously pointed out by Mr. Johnson in his Theory of fever. The distribution also of the nervous influence to the digestive organs, is as closely connected as the distribution of the circulating blood; for the semilunar ganglion, forming the great central plexus of nerves which surrounds the root and branches of the cœliac artery, being derived from the great sympathetic, and connected also with the par vagum, sends its communicating branches to the liver, spleen, stomach, pancreas, and duodenum. Be it remembered also, that the nerves which pass to the lower orifice of the stomach and duodenum, are branches of the right hepatic plexus, derived from the same origin at the cœliac union. We can easily see then, how any distending cause which irritates any one of these viscera will readily affect the others, independent of mechanical pressure from obstruction to the circulating blood; and how all will be speedily brought into a morbid condition by a continued affection of any one of them. And as the energy derived from the sensorium concentrates in the connecting medium of the cœliac plexus common to all the digestive organs, the manner in which the whole of these viscera become morbidly affected by any injurious impression on the brain, is not difficult to be understood. 97.

We have thus divested Dr. Yeats's publication of much epistolary circumlocution, and exhibited its *essence* in a concentrated state to our readers. The author appears to be an observant, intelligent, and well-informed physician, and his letter is creditable to his talents and discrimination.

---

